# Interferon Regulator Factor 8 (IRF8) Limits Ocular Pathology during HSV-1 Infection by Restraining the Activation and Expansion of CD8^+^ T Cells

**DOI:** 10.1371/journal.pone.0155420

**Published:** 2016-05-12

**Authors:** Lin Sun, Anthony J. St. Leger, Cheng-Rong Yu, Chang He, Rashid M. Mahdi, Chi-Chao Chan, Hongsheng Wang, Herbert C. Morse, Charles E. Egwuagu

**Affiliations:** 1 Molecular Immunology Section, National Eye Institute (NEI), National Institutes of Health (NIH), Bethesda, Maryland, United States of America; 2 Immunoregulation Section, NEI, NIH, Bethesda, Maryland, United States of America; 3 Immunopathology Section, NEI, NIH, Bethesda, Maryland, United States of America; 4 Laboratory of Immunogenetics, National Institute of Allergy and Infectious Diseases (NIAID), NIH, Rockville, Maryland, United States of America; UC Irvine Medical Center, UNITED STATES

## Abstract

Interferon Regulatory Factor-8 (IRF8) is constitutively expressed in monocytes and B cell lineages and plays important roles in immunity to pathogens and cancer. Although IRF8 expression is induced in activated T cells, the functional relevance of IRF8 in T cell-mediated immunity is not well understood. In this study, we used mice with targeted deletion of *Irf8* in T-cells (IRF8KO) to investigate the role of IRF8 in T cell-mediated responses during herpes simplex virus 1 (HSV-1) infection of the eye. In contrast to wild type mice, HSV-1-infected IRF8KO mice mounted a more robust anti-HSV-1 immune response, which included marked expansion of HSV-1-specific CD8^+^ T cells, increased infiltration of inflammatory cells into the cornea and trigeminal ganglia (TG) and enhanced elimination of virus within the trigeminal ganglion. However, the consequence of the enhanced immunological response was the development of ocular inflammation, limbitis, and neutrophilic infiltration into the cornea of HSV-1-infected IRF8KO mice. Surprisingly, we observed a marked increase in virus-specific memory precursor effector cells (MPEC) in IRF8KO mice, suggesting that IRF8 might play a role in regulating the differentiation of effector CD8^+^ T cells to the memory phenotype. Together, our data suggest that IRF8 might play a role in restraining excess lymphocyte proliferation. Thus, modulating IRF8 levels in T cells can be exploited therapeutically to prevent immune-mediated ocular pathology during autoimmune and infectious diseases of the eye.

## Introduction

Interferon regulatory factor 8 (IRF8), also known as ICSBP (interferon consensus sequence-binding protein), is a transcription factor that is primarily expressed in cells of the immune system [1]. Similar to the other 8 members of the interferon regulatory factor (IRF) family of transcription factors, IRF8 is characterized by an N-terminal DNA-binding domain (DBD) that mediates binding to the IFN-stimulated response element (ISRE) and a C-terminal IRF-association domain (IAD), which facilitates dimerization with other members of the IRF family as well as ETS family members [1, 2]. IRF8 can repress or activate gene transcription depending on the specific DNA recognition sequence prescribed by its interacting partner [1, 2]. It is constitutively expressed in monocytes and B cell lineages and plays important roles in host immunity to pathogens. IRF8 regulates B cell differentiation and plays key regulatory roles in the development and functional maturation of microglia, mast cells, basophils and dendritic cells [3–5]. While expression of IRF8 is rapidly induced in T cells in response to TCR activation and/or cytokine stimulation, the role of IRF8 in the development or effector functions of T cells is less well understood [6]. However, recent studies in mice indicate that IRF8 directs a silencing program for Th17 differentiation through its physical interaction with the Th17 master transcription factor, RORγt and promotes neuroinflammation by activating integrin-mediated TGF-β signaling [7, 8]. In this study, we sought to understand the role of IRF8 in cell-mediated immunity to ocular HSV-1 infection.

Herpes simplex virus type 1 (HSV-1) is a prevalent pathogen of humans and a variety of animal species with more than half of the human population infected with HSV-1 by age 70 [9]. Primary HSV-1 infection of the eye results in the colonization of many sensory neurons of the trigeminal ganglion (TG) with the viral genome persisting in a quiescent state as episomal DNA in neurons [10, 11]. The latent virus can persist in neurons throughout the life of the host and although viral lytic gene products are produced intermittently without virus production, CD8^+^ T cells surrounding latently infected TG neurons are thought to block HSV-1 reactivation and subsequent disease [10–12]. Nonetheless, occasional reactivation of the virus in neurons and its transport to the ocular surface tends to elicit immune responses in the cornea. Repeated reactivation events can cause progressive and recurrent scarring of the cornea, which may lead to the blinding form of the disease, herpetic stromal keratitis (HSK). As HSK is the leading cause of infectious blindness in developed countries, there is significant interest in immunological mechanisms that regulate ocular HSV-1 infection and the maintenance of HSV-1 latency in TG.

In this study, we used mice that lack IRF8 in T cells (IRF8KO) to examine whether IRF8 mediates transcription of genes that regulate anti-viral activities of T cells. We observed significant increases in HSV-1-specific CD8^+^ T cell responses locally in the TG as well as peripherally in the draining lymph nodes and spleen, resulting in more effective viral clearance. The data are discussed in context of the role of IRF8 in the development of effector and memory CD8^+^ T cell responses and potential use of IRF8 to mitigate ocular pathology.

## Methods and Materials

### Animals and reagents

C57BL6/J and C57BL6/JCD45.1, and CD8αKO mice (6–8 weeks old) were purchased from Jackson Laboratory (Bar Harbor, ME, USA). CD4-STAT3O mice were generated in house [13]. We derived mice with conditional deletion of *Irf8* in T cells (IRF8KO) by breeding Irf8^fl/fl^ mice with CD4-Cre (Taconic, Hudson, NY) mice. Littermate Irf8^fl/fl^ mice on the C57BL/6J background, were used as wild type (WT) controls. Mice were maintained and used in accordance with NEI/NIH Animal Care and Use Committee guidelines (Study # EY000262-19 & EY000372-14). For analysis of HSV-1-specific responses, the HSV-1 gB (498–505, SSIEFARL/PE) peptide used was synthesized and HPLC-purified by Invitrogen. H-2K^b^ HSV-1 gB-Tetramers were synthesized by NIH Tetramer facility, Emory Univ., Atlanta, GA. National Institutes of Health (NIH) Animal Care and Use Committee approved the study protocol used in studies described in this manuscript (ASP # NEI-697).

### Herpes Simplex Virus 1 (HSV-1) infection

Recombinant HSV-1 virus, RE-pICP0-EGFP, was kindly provided by Paul R. Kinchington, (University of Pittsburgh, School of Medicine, Pittsburgh, Pennsylvania). The virus was propagated on Vero cell monolayers and purified as previously described [14]. Briefly, confluent monolayers of Vero cells were infected with HSV-1 at m.o.i (multiplicity of infection) of 0.01. After 2h adsorption, virus was aspirated and the monolayers were washed once with serum-free medium and then refed with fresh serum-free medium. The cells were further incubated at 37°C. At maximum cytopathic effect, supernatant was collected and the virus was further purified by sucrose density-gradient centrifugation (10–60% w/v) for 1h at 11,500 rpm using a Beckman SW28 rotor or by OptiPrep gradients super-centrifugations according to the manufacturer’s instructions (Accurate Chemical & Scientific Corp., Westbury, NY). Infectivity of the purified virus was determined by plaque titration with the methylcellulose-overlay method. In brief, Vero cell monolayers on a 24-well plate were infected with a series of diluted virus suspensions for 2h, after which the monolayer was washed once with fresh serum-free medium supplemented with 2% FBS. Forty-eight hours post-infection (p.i.), viral plaques were counted and titrated. Virus was further purified by sucrose density-gradient centrifugation (10–60% w/v) using a Beckman SW28 rotor for 1h at 11,500 rpm or by OptiPrep gradients super-centrifugations according to the manufacturer’s instructions (Accurate Chemical & Scientific Corp., Westbury, NY). C57BL/6 mice (WT), IRF8KO, CD4-STAT3KO and CD8αKO (10 mice per group) were infected with 2x10^5^ plaque-forming units (pfu)/mouse by scarification of the corneal epithelial layers (18 times). For intraperitoneal inoculation, mice were anesthetized by intramuscular injection of ketamine hydrochloride, and virus in 100 μl DMEM was injected using a 30G needle. Two days after inoculation, spleen cells were isolated and analyzed by FACS.

### Histology

Eyes were carefully enucleated from HSV-1-infected mice, fixed in 4% formaldehyde for 30 min and transferred to 10% buffered formalin for over 24 hours. Specimens were dehydrated through graded alcohols and embedded in paraffin. Serial vertical sections through the pupillary-optic nerve plane were cut and stained with hematoxylin and eosin (H&E). Photographs of representative sections were taken on an Olympus photomicroscope.

### FACS analysis and intracellular protein staining

Freshly isolated peripheral blood mononuclear cells (PBMC), lymph node (LN) or spleen cells were subjected to cell surface FACS analysis using the labeled mAbs indicated on the figures. CD8^+^ and CD4^+^ T cells were enriched by negative selection on CD3-cell-enrichment column. The cells were further purified using anti-CD4 or anti-CD8-Ab conjugated magnetic beads (Miltenyi). In some experiments, gB-tetramer positive CD8 T cells were sorted electronically on a FACSAria II sorter (BD bioscience, CA). For analysis of cells in the trigeminal ganglion or the cornea of HSV-infected mice, the mice were anesthetized, perfused and the isolated trigeminal ganglia (TG) or minced corneas were digested with collagenase for 1 hour at 37°C, and then subjected to FACS analysis. The intracellular cytokine-staining assay was performed as previously described [15]. Briefly, the cells were re-stimulated with PMA (20ng/ml) and ionomycin (1μM) for 5 hours, and brefeldin A (5 μg/ml) was added during the last hour. The intracellular cytokine staining assay was performed in a Becton-Dickinson FACSCalibur using BD Biosciences Cytofix/Cytoperm kit as recommended (BD Pharmingen, San Diego, CA).

### Lymphocyte proliferation

Purified naïve CD8^+^ T cells were cultured for 4 days under non-polarizing condition. After 36 h, cultures were pulsed with ^3^H-thymidine (0.5 μCi/10 μl/well) for 12 additional hours and analyzed as described [16]. Data are mean CPM ± S.E. of responses of five replicate cultures.

### CD8^+^ T cell adoptive transfer

Lymphocytes were isolated from wild and IRF8KO mice. The T-lymphocytes were isolated by negative selection by using a mouse CD3 T cell enrichment column kit (R & D Systems). CD8^+^ T cells were then purified by CD8a-Microbeads by magnetic sorting. The purity of CD8^+^ T cells was more than 96%. The cells were counted by using Vi-Cell viability analyzer (Beckman Coulter) and viability of the cells used for adoptive transfer was more than 98%. Adoptive transfer of the wild type or IRF8KO CD8^+^ T cells (10x10^6^) was by tail vein injection. Two days after transfer, the mice were infected with HSV-1 as described early and 8 days post infection, the mice were euthanized for immunological assays and virus detection.

### Quantifying live virus from the ocular surface

On day 6 p.i., the corneal surface of each eye was gently swabbed with sterile Weck-Cel surgical spears (Medtronic Solan Jacksonville, FL). Swabs from right and left eyes were added separately, in duplicate, to a 95% confluent layer of Vero cells, incubated 1 hr at 37°C, and overlayed with plaque assay media. The cultures were incubated for 48 hrs, fixed with formalin, and stained with crystal violet. A dissecting microscope was used to detect viral plaques.

### RT-PCR and quantitative and semi-quantitative RT-PCR analysis

Total RNA was extracted using the Trizol reagent according to the procedures recommended by the manufacturer (Life Technologies, Gaithersburg, MD). RNA integrity was verified by analysis of 18S and 28S ribosomal RNA expression on RNA gels. RNA (10 μg), SuperScript III Reverse Transcriptase (Life Technologies, Gaithersburg, MD), and oligo (dT)_12–16_ were used for first-strand synthesis as previously described [17]. All cDNA preparations used were suitable substrates for PCR amplification on the basis of efficient amplification of a β-actin sequence and each gene-specific primer pair used for RT-PCR analysis spans at least an intron. Real-time PCR was performed on an ABI 7500 (Applied Biosystems) and PCR parameters were as recommended for the TaqMan Universal PCR kit (Applied Biosystems). Primers and probes were purchased from (Applied Biosystems). The relative mRNA expression levels were normalized to the levels of the housekeeping gene, *β-actin*, or *gapdh*. Genomic DNA was isolated from the TG using Wizard Genomic DNA Purification Kit (Promega, WI, USA). DNA specific to the glycoprotein H (gH) gene of HSV-1 was amplified using the RT-SYBR Green ROX qPCR mastermix was purchased (QIAGEN, MD, USA). The qPCR primer sequences are 5’-CGAACAGAGAGCTTGGCATTC-3’ and 5’-TGAGGTAGGTTAGGAACATG-3’.

### Western blot analysis

Whole cell lysates were fractionated on a 4–12% gradient SDS-PAGE and transferred to nitrocellulose membranes. Antibodies used are specific to mouse IRF8 or β-Actin (Santa Cruz Biotechnology). Pre-immune serum was used in parallel as controls and signals were detected with HRP conjugated-secondary F(ab')2 Abs (Zymed Labs, San Francisco, CA) using the ECL Plus Kit (Amersham, Arlington Heights, IL).

### Statistical analyses

Statistical analyses were performed by independent two-tailed Student’s t-test. Data presented as mean + SD. Asterisks denote p value (*P < 0.05, **P < 0.01, ***P < 0.001, ****P < 0.0001).

## Results

### IRF8-deficient T cells exhibit a pre-activated phenotype and exhibit higher proliferative capacity in responses to antigenic stimulation

**Fig 1:**
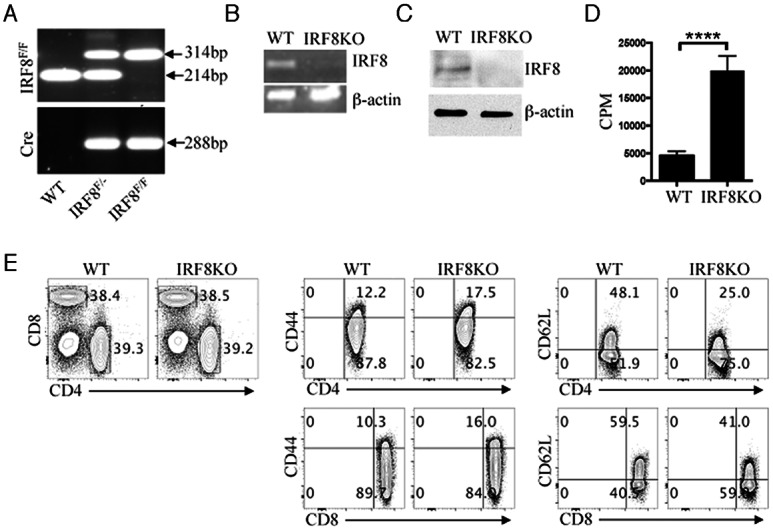
IRF8-deficient T cells exhibit a pre-activated phenotype and more proliferative capacity in response to antigenic stimulation. (A) IRF8^fl/fl^ mice were cross-bred with CD4-Cre mice to generate mice with deletion of IRF8 in the CD4 and CD8 compartments (IRF8KO) and the IRF8^fl/fl^/Cre alleles were identified by PCR analysis of mouse tail genomic DNA. Presence of the 314bp band indicates *Irf8*-floxed DNA, while the 214bp band is consistent with size of the endogenous *Irf8* gene sequence in the wild-type mouse genome. (B, C) Naïve CD8^+^ T cells isolated from the spleen were activated with anti-CD3/CD28 for 3 days and analyzed for IRF8 expression by (B) RT-PCR or (C) western blotting. (D) The TCR-activated WT or IRF8KO CD8^+^ T cells were also analyzed by the thymidine incorporation assay. (E) Naïve T cells isolated from spleen were analyzed by FACS. Numbers in quadrants indicate percentages of T cells expressing the cell surface markers, CD44 or CD62L. Data represent at least 3 independent experiments.

CD8^+^ T cells play important roles in host immunity against viruses and mediate anti-viral activities by differentiating into short-lived cytotoxic T lymphocytes (CTLs) characterized by expression of high levels of KRLG-1 (killer lectin-like receptor subfamily G member 1) and IFN-γ [18, 19]. In this study, we generated mice with targeted deletion of *Irf8* in T cells to investigate the role of IRF8 on immune responses to HSV-1. We generated the IRF8KO mouse strain by mating CD4-Cre and *Irf8*^*fl/fl*^ mouse strains on C57BL/6J background [20] and each mouse used in this study was genotyped by PCR analysis of tail DNA (Fig 1A). In addition, we confirmed that *Irf8* was indeed deleted in IRF8KO T cells by RT-PCR in CD8^+^ T cells (Fig 1B) or western blot (Fig 1C). We then examined whether IRF8 regulates CD8^+^ T cell proliferation by stimulating sorted CD8^+^ T cells for 4 days with anti-CD3/CD28 Abs and subjecting the cells to a thymidine incorporation assay. Result of thymidine incorporation analyses indicates a significant increase in the proliferative response of IRF8KO CD8^+^ T cells in comparison to their WT counterpart (Fig 1D), suggesting that a physiological role of IRF8 in CD8^+^ T cell might be to prevent excessive proliferation. We also found that IRF8KO T cells exhibited an enhanced activation phenotype as indicated by increased frequencies of CD44-expressing T cells and reduced frequencies of T cells expressing high levels of CD62L in the spleens of IRF8KO mice (Fig 1E).

### Loss of IRF8 in T cells correlates with enhanced ocular inflammation during HSV-1 infection

**Fig 2:**
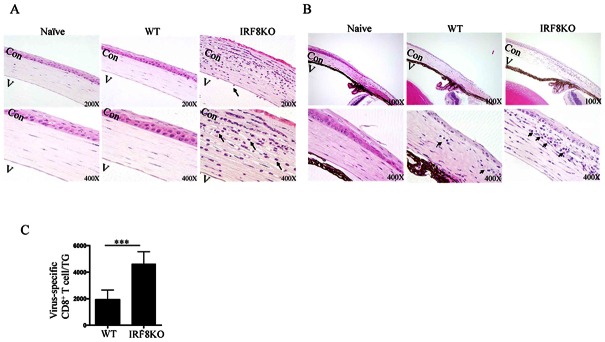
Loss of IRF8 in T cells correlates with ocular inflammation during HSV-1 infection. WT (C57BL/6J) or IRF8KO mice were infected with HSV-1. (A & B) Inflammation was determined by histology. For histological analysis, eyes were harvested on day 8 post-infection (p.i.) and sections through the (A) cornea or (B) limbus were stained with hemotoxylin and eosin and depicted inflammatory cellular infiltration (arrows). Naïve: naïve WT, AC:anterior chamber, Cor: corneal. (C) Trigeminal ganglia (TG) were isolated on day 8 p.i., digested with collagenase and analyzed by flow cytometry. The cells were gated on CD45 and absolute numbers of gB-Tetramer^+^ CD8^+^ T cells were determined. Statistical analysis of the virus titer was based on analysis of 5 mice per group. Data represent at least 3 independent experiments.

We next examined whether IRF8 plays a role in T cell-mediated regulation of viral infection. We infected the ocular surface of WT or IRF8KO mice with the recombinant REpICP0-EGFP HSV-1 strain [21] and found that the infection was cleared by day-8 p.i. with very little or no ocular pathology in the WT mice (Fig 2A). In contrast, the IRF8 KO mice developed corneal inflammation and limbitis (Fig 2A and 2B) and correlated with increase of gB_498-505_-specific CD8^+^ T cells within the TG (Fig 2C) [22, 23]). It is of note that the observed increase in CD8^+^ T cells in TG is consistent with previous reports showing that HSV-specific CD8^+^ T cell responses peak at ~8 DPI within the TG, surround latently infected neurons, and confer protection against viral reactivation [11].

**Fig 3:**
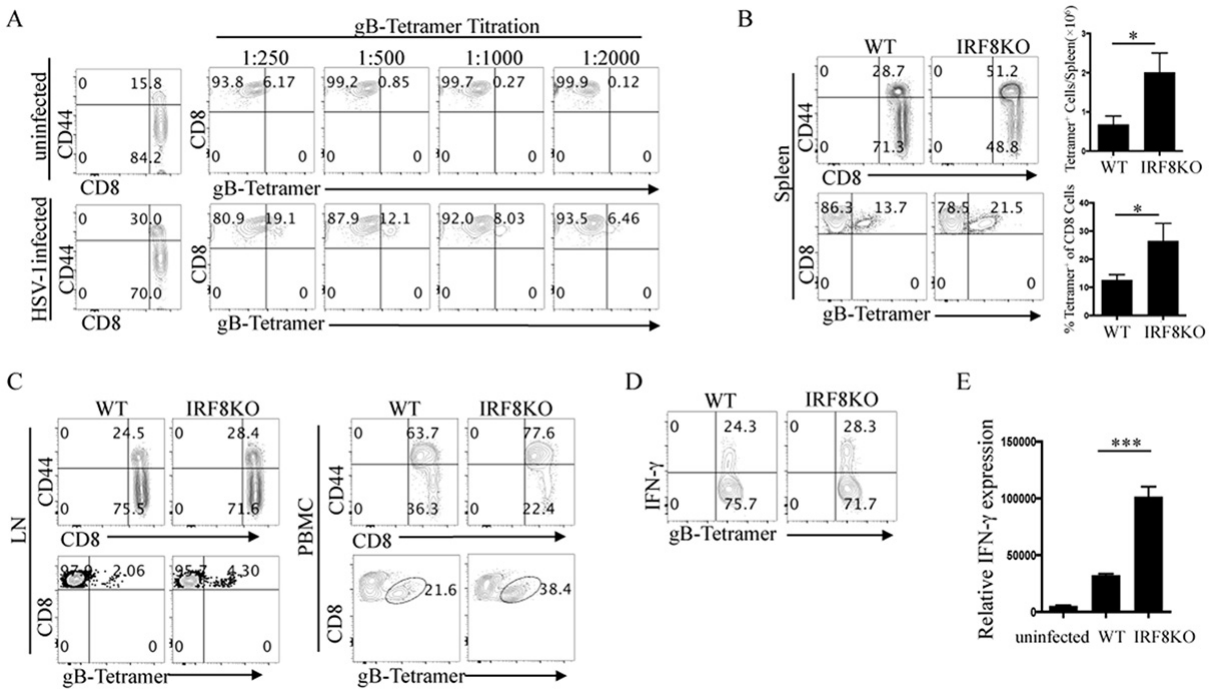
IRF8-deficient T cells are more activated during ocular HSV-1 infection. (A) WT mice were challenged with HSV-1 by corneal scarification and we titrated the gB-tetramer to analyze and characterize the virus-specific CD8^+^ T cell responses in the spleen. (B-D) IRF8KO and WT mice were infected with HSV-1 by corneal scarification and cells isolated from the (B) spleen, or (C) PBMC (C, D) LN on day 8 p.i were analyzed by FACS using the gB-tetramer. The cells were gated on CD3/CD8 and numbers in quadrants indicate percentages of CD8^+^ T cells expressing CD44 and CD8^+^ gB-tetramer^+^ T cells expressing IFN-γ. (E) IFN-γ expression by CD8^+^ T cells was analyzed by real-time qPCR. Data represent at least 3 independent experiments.

To investigate the role of IRF8 in regulating the expansion of CD8^+^ T cells *in vivo*, we infected IRF8KO and WT mice with HSV-1 and analyzed the relative levels of HSV-1-specific (gB-tetramer^+^) CD8^+^ T cells [22, 24] in the spleens, LNs and blood. Dose-dependent titration of the tetramer established that a 1:1000 dilution of the tetramer is the optimal concentration for detecting HSV-1-specific CD8^+^ T cells (Fig 3A) and this concentration was used in all subsequent analyses. We observed significant increases in the percentages and/or absolute numbers of HSV-specific CD8^+^ T cells in the spleen, LN and blood of IRF8KO mice (Fig 3B and 3C), suggesting that IRF8 inhibits CD8^+^ T cell activation and proliferation *in vivo*. Analysis of HSV-1-specific CD8^+^ T cells in the spleen showed modest increase in the percentages of IFN-γ-producing cells and IFN-γ mRNA transcripts in the spleens of IRF8KO compared to WT mice (Fig 3D and 3E). However, it is unclear whether IRF8 has a direct effect on *Ifng* gene transcription or if its effect is indirect as a consequence of across-the-board inhibition of the proliferation of all T cell subsets. Taken together these results suggest that a consequence of the exuberant anti-virus response mounted by the IRF8KO mice may be the induction of ocular inflammation and limbitis during primary infection.

### IRF8 regulates expression of chemokine receptors and integrins in CD8^+^ T cells

**Fig 4:**
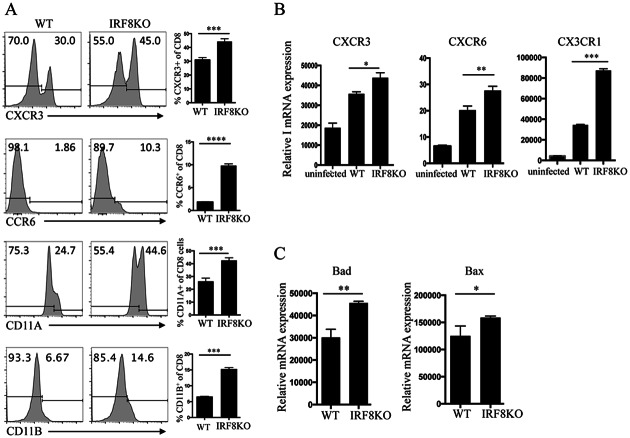
IRF8 regulates expression of chemokine receptors and integrins in CD8^+^ T cells. (A) IRF8KO and WT mice were infected with HSV-1 and on day 8 p.i. We isolated CD8^+^ T cells from the spleen and analyzed their immunophenotype by flow cytometry. Numbers on the histograms indicate percentage of CD8^+^ T cells expressing CXCR3, CCR6, CD11a or CD11b. (B,C) RNA isolated from sorted CD8^+^ T cells were analyzed by qPCR for the expression of (B) chemokine receptors, (C) pro-apoptotic molecules. Data represent at least 3 independent experiments.

Recent studies have established that CD8^+^ T cells are rapidly activated and recruited into the TG in response to ocular HSV-1 infection and that their presence in the TG is temporally associated with elimination of replicating virus from ganglions [25, 26]. We therefore examined whether the observed increase in the recruitment of HSV-1-specific CD8^+^ T cells into TG of infected IRF8KO mice derived from aberrant regulation of the expression of chemokine receptors or integrins that mediate trafficking of T cell to inflammatory foci. We infected IRF8KO and WT mice with HSV-1, isolated CD8^+^ T cells from spleens (day 8 p.i) and FACS analysis of the cells revealed significantly higher levels of CXCR3, CCR6, CD11a and CD11b on IRF8KO CD8^+^ T cells compared to cells from WT mice (Fig 4A). Analysis of RNA isolated from the cells also showed significant increases in transcription of CXCR3 and CXCR6 (Fig 4B) as well as genes that regulate T-lymphocyte survival (Fig 4C). It is however interesting that IRF8-deficient Treg cells were defective in CXCR3 expression [27], suggesting that IRF8 might have differential effects on regulatory and effector T cell subsets. Nonetheless, as CXCR3, CCR6, CD11a and CD11b mediate lymphocyte trafficking, these results suggest that another function of IRF8 in CD8^+^ T cells might be to inhibit trafficking of inflammatory cells into the eye, thereby restraining excessive inflammatory responses in this immune privileged tissue [23].

### IRF8 regulates the expansion of memory precursor effector cells (MPECs)

**Fig 5:**
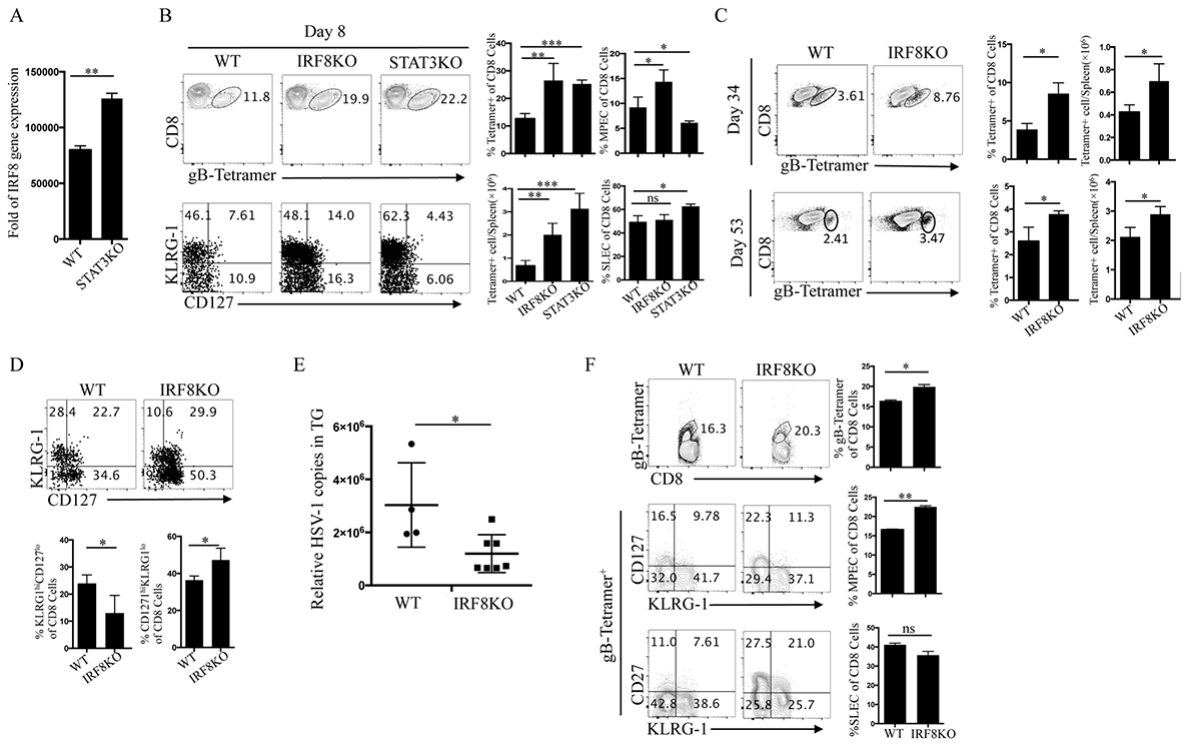
IRF8 regulates the expansion of memory precursor effector cells (MPECs). (A) WT or STAT3KO mice were infected with HSV-1 and RNA from sorted CD8^+^ T cells on day 8 p.i were analyzed by qPCR for IRF8 expression. (B-D) WT or CD4-IRF8KO mice were infected with HSV-1 and CD8^+^ T cells were isolated from the spleen on day (B) 8 p.i, day (C) 34 or day 53 p.i and analyzed by FACS. (B) CD8^+^ T cells were first gated on gB-Tetramer+ population and then the percentages of MPEC (KLRG-1^lo^CD127^hi^) and SLEC (KLRG^hi^CD127^lo^) in gB-Tetramer^+^ population were analyzed. (D) Flow plots represent the total gB-tetramer positive T cell population. The numbers in quadrants indicate percentages of CD8^+^ gB-tetramer-positive T cells expressing KLRG-1 and/or CD127. (E) Genomic DNA extracted from TG were analyzed by qPCR of the HSV-1 genes. (F) WT and CD4-IRF8KO mice were also infected with HSV-1 by the intraperitoneal route (i.p.) and CD8^+^ T cells were isolated from the spleens of infected mice on day 6 p.i, gated on CD8^+^gB-tetramer-positive and numbers in quadrants indicate percentages of T cells expressing KLRG-1 and/or CD127. Data represent at least 3 independent experiments.

Acute viral infection induces naive CD8^+^ T cells to differentiate into short-lived effector cells (SLEC) or memory-precursor effector cells (MPEC), which are distinguishable by the levels of cell surface expression of KLRG-1 (Killer cell lectin-like receptor subfamily G member 1) and CD127 [28]. KLRG-1 is a trans-membrane protein expressed in response to TCR activation following presentation of virus epitopes in context of MHC class I molecules and is a marker of virus-specific CD8^+^ T cell responses while CD127, also known as IL-7 receptor α chain (IL-7Rα), is a useful marker for identifying memory T cells [28–30]. Recent studies have shown that the STAT3 signaling pathway is critical for functional maturation of memory CD8^+^ T cells and IRF8 is a novel target of STAT3 [31, 32], suggesting that a STAT3/IRF8 axis may regulate T cell differentiation. We show here that the expression of IRF8 is upregulated in STAT3KO CD8^+^ T cells (Fig 5A), suggesting that STAT3 might negatively regulate IRF8 in T cells. To examine whether IRF8 and STAT3 exert mutually antagonistic effects on the maturation of memory CD8^+^ T cell, we infected WT, IRF8KO and STAT3KO mice with HSV-1 and analyzed the levels of MPEC (KLRG-1^lo^CD127^hi^) or SLEC (KLRG-1^hi^CD127^lo^) CD8^+^ T cells in the spleen. During the acute immune response (day 8 p.i.), we observed significant expansion of virus-specific CD8^+^ T cells in the spleens of IRF8KO and STAT3KO mice as compared to spleens of WT mice (Fig 5B). Consistent with a previous report indicating the requirement of STAT3 for maturation of memory CD8^+^ T cells, the percentages of MPEC in STAT3KO spleens was significantly lower than the levels in spleens of WT mice. In contrast, the percentages of MPEC in spleens of IRF8KO mice was significantly higher than the levels in spleens of WT mice (Fig 5B), suggesting that IRF8 and STAT3 might have diametrically opposed effects on the maturation of CD8^+^ memory T cells. On the other hand, while the loss of STAT3 in CD8^+^ T cells suppressed the expansion of SLEC, IRF8-deficiency did not affect the level of SLEC (Fig 5B). We also determined the percentages of virus-specific CD8^+^ T cells in the spleens at 34 and 53 days p.i. In contrast to our findings on day 8 p.i, the percentages of virus-specific CD8^+^ T cells in the spleens of IRF8KO mice 34 or 53 days p.i. was significantly lower than the frequencies in spleens of WT mice (Fig 5C). Nonetheless, by day 53 p.i., the frequencies of MPEC increased significantly in the spleens of IRF8KO compared to WT mice, further indicating that IRF8 suppresses maturation of CD8^+^ memory T cells during ocular HSV-1 infection (Fig 5D). In fact, the increased frequencies of MPEC correlated with a significant reduction of viral load in the TG of day 53 IRF8KO mice compared to their WT counterparts (Fig 5E). Together, our data suggest that the enhanced anti-viral T cell response seen in IRF8KO mice might facilitate a more effective clearance of HSV-1 from the host. Because of the unique features of ocular immune privilege, it is possible that the effects of the loss of IRF8 on the maturation of CD8^+^ memory T cell may only pertain to HSV-1 infection in the cornea. We therefore infected WT and IRF8KO mice with HSV-1 by the intraperitoneal route. We isolated CD8^+^ T cells from the spleens on day 6 p.i, and determined the relative abundance gB-Tetramer-positive CD8^+^ MPEC. Consistent with findings in mice infected by corneal scarification, significantly higher percentages of MPEC were also observed in the spleens of IRF8KO versus spleens of WT mice (Fig 5F).

### Adoptive transfer of CD8^+^ deficient T cells reduced viral load and enhanced viral-induced inflammation in HSV-1-infected mice

**Fig 6:**
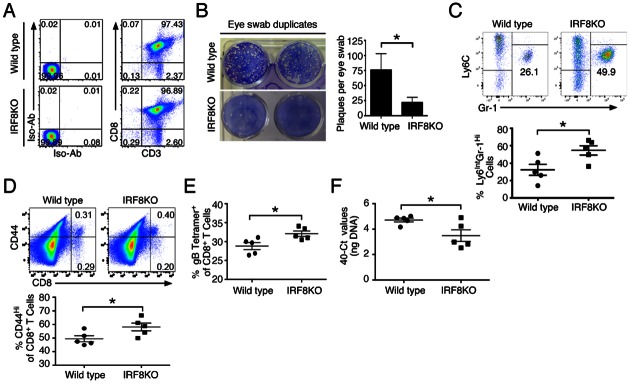
Adoptive transfer of CD8^+^ deficient T cells reduced viral load and enhanced viral-induced inflammation in HSV-1-infected mice. (A) CD8^+^ T cells from WT or IRF8 KO mice were MACS sorted and 10 x 10^6^ cells were transferred into CD8αKO mice. After 1 day, mice were ocularly infected with 2 x 10^5^ pfu of HSV-1 via scarification. (B) On day 6 p.i., eye swabs were taken and infectious virus was assessed by plaque assay in duplicates (N = 20). Bars represent the mean pfu ± SEM of virus per cornea. Significance was determined by Mann-Whitney U test. (C-F) Mice were then sacrificed on day 8 p.i. (C) Corneas were harvested, digested with collagenase and inflammatory cells recruited to the cornea were assessed by flow cytometry. Bars represent the mean frequency ± SEM of Ly6C^Int^Gr-1^hi^ neutrophils from the CD11b^+^ gate. Flow plots represent the quantity of cells within the cornea. (D & E) Draining lymph nodes were harvested and activated CD8^+^ T cells (D) and virus-specific (gB-positive) CD8^+^ T cells (E) were analyzed. The cells were gated on CD8^+^ population and numbers in the quadrants of the FACS plot represent the frequency of CD44^hi^ cells while bars on the graphs represent the mean frequency ± SEM of CD44^hi^ or gB-tetramer specific CD8^+^ T cells. (F) DNA from the TG was harvested (day 8 p.i.) and qPCR was performed with HSV-1-specific primers. Bars in the graph represent the mean DNA level ± SEM of 5 mice.

Although it is well-established that CD8^+^ cytotoxic cells play dominant roles in anti-HSV-1 immune responses [21, 33], we sought to demonstrate directly that the enhanced clearance of HSV-1 from the host and the resulting immune-mediated ocular inflammation were induced as a result of the loss of IRF8 in CD8^+^ T cells. We isolated WT or IRF8 KO CD8^+^ T cells (Fig 6A) and adoptively transferred them into CD8α KO mice. Mice were then infected by inoculation of HSV-1 (2x10^5^ pfu) per eye. At day 6 p.i., we observed a more efficient elimination of live virus at the corneal surface of mice that received IRF8 KO CD8^+^ T cells (Fig 6B). Although clearance of HSV-1 from eyes of C57BL/6 mice usually occurs by ~day 5–6 pi, we surmised that clearance of the virus might be delayed in the CD8α KO recipient mice that lack a functional CD8 compartment. We therefore extended our analysis to day 8 p.i. Interestingly, both groups of mice had inflammatory cell infiltration of the cornea at day 8 p.i. In agreement with our earlier results (Fig 2), mice that were received IRF8 KO CD8^+^ T cells developed increased corneal inflammation as observed by increased frequency of Ly6C^int^Gr-1^hi^ neutrophils (Fig 6C) compared to CD8α KO mice supplemented with WT CD8^+^ T cells. Corroborating the results from our studies in the IRF8 KO mice, we observed a significant increase in activated HSV-1-specific (gB-Tetramer^+^) CD8^+^ T cells within the DLN (Fig 6D and 6E). We also observed significant reduction of HSV-1 DNA in the TG at day 8 p.i. (Fig 6F) that correlates with significant expansion of activated HSV-1-specific CD8^+^ T cells in the TG (Fig 2C). Taken together these results underscore the role of IRF-8-expressing CD8^+^ T cells in restraining inflammation and suggesting that the loss of IRF8 in CD8^+^ T cells might promote a stronger anti-viral response leading to a more efficient clearance of HSV-1 in the eye.

## Discussion

In this study, we used mice with a targeted deletion of *Irf8* in the T cell compartment (IRF8KO) to characterize the role of IRF8 in CD8^+^ T cells. Specifically, we examined whether the loss of IRF8 would affect the maturation of CD8^+^ T cells and CD8^+^ lymphocyte-mediated host immunity against viruses. We show here that IRF8-deficient CD8^+^ T cells divided faster than WT CD8^+^ T cells in response to TCR stimulation and exhibited enhanced proliferative capacity. Thus, following infection of IRF8KO and WT mice with HSV-1 by corneal scarification or intraperitoneal injection, the IRF8KO mice mounted a more vigorous immune response characterized by increased numbers of virus-specific CD8^+^ T cells. In addition, markedly elevated levels of CXCR3, CXCR6 and CCR6 on the IRF8-deficient CD8^+^ T cells correlated with increased infiltration of CD8^+^ T cells into the TG of the IRF8KO mice. It is important to note that our IRF8KO mice lack IRF8 in CD4^+^ as well as CD8^+^ T cells. We therefore isolated WT or IRF8 KO CD8^+^ T cells (Fig 6A) and adoptively transferred them into HSV-1-infected mice that lack CD8^+^ T cells (CD8α KO). Mice that received IRF8KO CD8^+^ T cells more efficient eliminated the virus in the eye (Fig 6B). Thus, our adoptive transfer experiment recapitulated essential features observed in the IRF8KO mice, suggesting that the enhanced anti-viral response and efficient elimination of HSV-1 noted in the eye of IRF8KO mice is largely attributable to loss of IRF8 in CD8^+^ T cells. Taken together, these observations suggest that IRF8 may play a role in regulating the transcription of genes that mediate the trafficking of CD8^+^ T cells to sites of inflammation. It is therefore of note that we observed increases of monocytes and neutrophils in the cornea of mice that received IRF8KO CD8^+^ T cells and this is particularly relevant in context of mechanisms that mediate virus clearance in the eye. In fact, neutrophils are known to be the primary cell subset involved in acute virus clearance from the eye and studies have shown that IFN-γ enhances the activation [34] and prolongs survival [35] of neutrophils. It is also of note that IRF8 is one of the immediate early genes induced by IFN-γ that may serve as a feedback regulator of transcriptional activities of this pro-inflammatory cytokine [36, 37].

IRF8 has previously been shown to regulate myeloid cell differentiation, maturation and lineage commitment [38]. IRF8 is a target of STAT3 [31, 32] and the STAT3 transcription factor plays critical roles in the functional maturation of memory CD8^+^ T cells [31]. We show here that transcription of *Irf8* gene is markedly elevated in STAT3KO CD8^+^ T cells, suggesting the existence of a STAT3/IRF8 regulatory axis in CD8^+^ T cells. While STAT3 signaling promotes the maturation and emergence of myeloid-derived suppressor cells (MDSCs), IRF8 overexpression attenuates MDSC accumulation and enhanced immunotherapeutic efficacy [32], suggesting that IRF8 and STAT3 signaling pathways may exert mutually antagonistic effects on T cell maturation and the establishment of T cell memory phenotype. It is however of note that a previous report using mice with global deletion of *irf8* showed that IRF8 suggested role for IRF8 in phenotypic maturation CD8 effector T cells and graft vs host disease (GvHD) [39]. However, dendritic cells from the global *irf8*-/- mice are defective in IL-12 production [40], making it difficult to know the extent to which IRF8-deficiency in other cell types might have contributed to the T cell developmental defects observed in those mice. We have circumvented these potentially confounding effects by selectively targeting *irf8* deletion in CD4^+^CD8^+^ double positive stage of T cell development in the thymus. Although, a dendritic cell subset does express CD4, the CD4 promoter in this DC subset is relatively weak and does not express sufficiently high levels of Cre to mediate deletion of *irf8*. This assertion is borne out by the universal use of CD4 Cre mice for specific gene in T cells [41]. We believe that our CD4-IRF8KO mice is an excellent model to further investigate the immunobiology of IRF8 in T cells and its varied roles in host immunity.

Ocular HSV-1 infection is of significant public health interest [14, 42]. Unlike HSV-1 infections of the skin or mucous membrane which are often non-progressive and mainly limited to epithelial lesions, ocular HSV-1 infection often involve the cornea (epithelium, keratocyte, endothelium), anterior uveal tract (iris, ciliary body), and/or the posterior segment (vitreous, retina, choroid, optic nerve) [14, 42]. HSV-1 infections that lead to herpetic stromal keratitis present unique challenges to the immune system because a very aggressive response to the pathogen can damage the visual axis while a tepid response may promote chronic persistent inflammation that can spread to the retina and induce retinal degenerative changes or retinal atrophy. During early HSV-1 infection, innate cells infiltrate cornea and eliminate replicating virus within corneal epithelia. Despite this immune response, HSV-1 still gains access to neurons and enters a latent state within the TG but viral-specific CD8^+^ T cells prevent viral reactivation. On the other hand, CD4^+^ T cells effector cells are thought to influence the development of corneal inflammation [43, 44]. It is possible that the increased T cell activation in the absence of IRF8 may allow CD8^+^ T cells to establish a more robust resident memory T cell population (T_RM_) at the ocular surface akin to the observations seen in skin model of HSV-1 infection [45], thereby better eliminating virus from the ocular surface.

In conclusion, our data provides insights into the role of IRF8 in T cell, particularly in the regulation of CD8^+^ T cell activation and development of CD8 memory phenotype. Our data further suggest that IRF8 expression may contribute to immune privilege of the eye by restraining exuberant inflammatory responses that produce cytotoxic cytokines that kill terminally differentiated retinal cells, leading to permanent ocular deficit or even vision loss.
